# S1P Signalling Axis Is Necessary for Adiponectin-Directed Regulation of Electrophysiological Properties and Oxidative Metabolism in C2C12 Myotubes

**DOI:** 10.3390/cells11040713

**Published:** 2022-02-17

**Authors:** Caterina Bernacchioni, Roberta Squecco, Tania Gamberi, Veronica Ghini, Fabian Schumacher, Michele Mannelli, Rachele Garella, Eglantina Idrizaj, Francesca Cencetti, Elisa Puliti, Paola Bruni, Paola Turano, Tania Fiaschi, Chiara Donati

**Affiliations:** 1 Department of Experimental and Clinical Biomedical Sciences “M. Serio”, University of Florence, 50134 Florence, Italy; caterina.bernacchioni@unifi.it (C.B.); tania.gamberi@unifi.it (T.G.); michele.mannelli@student.unisi.it (M.M.); francesca.cencetti@unifi.it (F.C.); elisa.puliti92@gmail.com (E.P.); paola.bruni@unifi.it (P.B.); tania.fiaschi@unifi.it (T.F.); 2 Section of Physiological Sciences, Department of Experimental and Clinical Medicine, University of Florence, 50134 Florence, Italy; roberta.squecco@unifi.it (R.S.); rachele.garella@unifi.it (R.G.); eglantina.idrizaj@unifi.it (E.I.); 3 Magnetic Resonance Center (CERM), University of Florence, 50019 Florence, Italy; ghini@cerm.unifi.it (V.G.); turano@cerm.unifi.it (P.T.); 4 Institute of Pharmacy, Freie Universität Berlin, Königin-Luise-Str. 2+4, 14195 Berlin, Germany; fabian.schumacher@fu-berlin.de

**Keywords:** adiponectin, sphingosine kinase, skeletal muscle, sphingosine 1-phosphate, electrophysiological properties, NMR metabolomics, oxygen consumption, S1P receptors

## Abstract

Background: Adiponectin (Adn), released by adipocytes and other cell types such as skeletal muscle, has insulin-sensitizing and anti-inflammatory properties. Sphingosine 1-phosphate (S1P) is reported to act as effector of diverse biological actions of Adn in different tissues. S1P is a bioactive sphingolipid synthesized by the phosphorylation of sphingosine catalyzed by sphingosine kinase (SK) 1 and 2. Consolidated findings support the key role of S1P in the biology of skeletal muscle. Methods and Results: Here we provide experimental evidence that S1P signalling is modulated by globular Adn treatment being able to increase the phosphorylation of SK1/2 as well as the mRNA expression levels of S1P_4_ in C2C12 myotubes. These findings were confirmed by LC-MS/MS that showed an increase of S1P levels after Adn treatment. Notably, the involvement of S1P axis in Adn action was highlighted since, when SK1 and 2 were inhibited by PF543 and ABC294640 inhibitors, respectively, not only the electrophysiological changes but also the increase of oxygen consumption and of aminoacid levels induced by the hormone, were significantly inhibited. Conclusion: Altogether, these findings show that S1P biosynthesis is necessary for the electrophysiological properties and oxidative metabolism of Adn in skeletal muscle cells.

## 1. Introduction

Skeletal muscle plays crucial functions in human metabolism and homeostasis, beyond the known contractile properties and the mechanical and structural functions of the organ. Skeletal muscle shows high plasticity to adapt to different stimuli, including hormones and nutrient levels [1]. Functional alterations of skeletal muscle are involved in multiple pathological conditions such as type-2 diabetes (T2D) [2] and Parkinson’s disease [3]. Skeletal muscle has also the ability to regenerate after a trauma by virtue of a specific population of muscle stem cells, named satellite cells. These cells, normally quiescent, are activated upon injury becoming able to proliferate and differentiate into skeletal muscle fibres [4].

Adiponectin (Adn) is one of the most concentrated hormones in the blood, accounting for 0.01% of total plasma proteins [5]. While Adn was initially considered a hormone produced exclusively by adipose tissue [6], and thus termed adipokine, it is now clear that it is locally secreted by different cell types such as skeletal muscle, osteoblast, placental and endothelial cells [7]. The secreted “full-length” form of Adn can be cleaved into smaller “globular” form or can assemble forming different multimers [8]. The different forms of Adn exert their biological functions binding to specific G-protein-independent, seven–transmembrane spanning receptors, AdipoR1 and AdipoR2. T-cadherin has been also identified as a receptor for high molecular weight Adn [9]. Adn is reported to increase insulin sensitivity in vivo both in liver and skeletal muscle [10], while a decrease of plasma Adn correlates with obesity and insulin resistance [11]. In skeletal muscle, Adn activates AMP kinase stimulating fatty acid oxidation and glucose uptake [12]. Beside its effects on energy homeostasis, globular Adn has been shown to induce skeletal muscle differentiation of C2C12 murine myoblasts through a redox-dependent circuitry involving p38, Akt and AMPK signalling [13]. Moreover, Adn has been proven to cause alterations of the resting membrane potential and membrane electrophysiological properties as well as of K^+^ and Ca^2+^ currents, inhibiting smooth muscle contractility of the gastric fundus [14].

Recently, it has been demonstrated that the ceramidase activity associated with AdipoR1 and AdipoR2, by enhancing the removal of fatty acyl chain from ceramides, is responsible for sphingosine generation that can subsequently be phosphorylated to sphingosine-1-phosphate (S1P), highlighting sphingolipid metabolism as a key element of Adn signalling pathway [15]. S1P is a bioactive lipid, normally present in plasma in submicromolar concentration, that regulates multiple fundamental processes, such as angiogenesis, lymphocyte trafficking and immune regulation [16]. S1P is synthesized from sphingosine by a phosphorylation reaction catalysed by two distinct isoenzymes sphingosine kinase (SK)1 and SK2 activated by different stimuli [17]. S1P can be reversibly converted to sphingosine by the action of specific and non-specific lipid phosphatases, while it is irreversibly degraded by S1P lyase [16]. S1P exerts its functions either as intracellular messenger or as a ligand of five specific G-protein coupled receptors named S1P receptors (S1PR) [18]. The release of S1P into the extracellular environment is mediated through the specific transporters spinster homolog2 (Spns2) [19] and Mfsd2b [20] or unspecific transporters belonging to the ATP-binding cassette (ABC) family [21].

In conditionally-inducible cardiac and pancreatic beta-cell specific model of apoptosis, Holland et al. [15] showed that inhibition of SK reversed the prosurvival effect of Adn in respect to palmitate- and C2 ceramide-induced cell death. In cardiac myocytes globular Adn is involved in mediating endothelial cell activation and behaves as physiological regulator of cyclooxigenase-2 signalling with a mechanism that depends on SK1: treatment with SK1 specific-siRNA reduced Adn-induced NF-kB activation and adhesion protein expression [22]. In ADN-KO mice restoration of Adn circulating levels reversed high fat diet-induced cardiac insulin resistance [23]. Moreover, treatment of H9C2 cardiomyoblasts and rat L6 skeletal muscle cells with AdipoRon, a small molecule agonist of AdipoR1 and AdipoR2, reduced palmitate-induced ROS production and lipotoxicity via the increase of S1P levels in the extracellular medium and activation of the S1P receptor-mediated signalling [23].

S1P plays a crucial role in the biology of skeletal muscle [24,25,26], acting as strong mitogenic [4,27] and promigratory cue in satellite cells [4] and inducing differentiation of myoblasts [28]. In murine “ex-vivo” skeletal muscles, S1P administration was shown to reduce the tension decline during fatigue [29]. Moreover, in C2C12 cells S1P/S1P_2_ signalling axis augmented ROS levels by a Rac1- and calcium-dependent mechanism, enhancing insulin sensitivity and glucose uptake [30]. In addition, exogenous S1P has been demonstrated to induce Ca^2+^ transients and cytoskeletal rearrangement in C2C12 myoblasts [31].

In the present study, we further investigated the biological action exerted by globular Adn in differentiated C2C12 skeletal muscle cells, highlighting new important regulatory effects of the hormone on active and passive electrophysiological properties as well as on cell oxidative metabolism. Notably, in both circumstances, the observed biological action driven by Adn was found to be mediated by S1P signalling axis, pointing at a critical role of this pathway in its action mechanism.

## 2. Materials and Methods

### 2.1. Materials

Mouse skeletal muscle C2C12 cells were obtained from the American Type Culture Collection (Manassas, VA, USA). All biochemicals, TRI reagent, cell culture reagents, Dulbecco’s Modified Eagle Medium (DMEM), foetal bovine serum (FBS), horse serum (HS), protease inhibitor cocktail, bovine serum albumin (BSA), D-erythro-MAPP, specific anti-Spns2 (N-TERM) antibodies and the specific SK1 inhibitor PF543 were purchased from Merck Life Sciences (Burlington, MA, USA). Murine recombinant globular adiponectin (gAcrp30) was purchased from BioVision Inc. (Milpitas, CA, USA). Selective SK2 inhibitor ABC294640 was obtained from Cayman Chemical (Ann Arbor, MI, USA). Anti-SK2 (N-terminal region), anti-SK1 (central region), anti-phospho-SK2 (Thr578) and anti-phospho-SK1 (Ser225) antibodies were purchased from ECM Biosciences LLC (Versailles, KY, USA). Enhanced chemiluminescence reagents was obtained from GE Healthcare Europe (Milan, Italy). Monoclonal anti-GAPDH-antibodies, as well as secondary antibodies conjugated to horseradish peroxidase, were obtained from Santa Cruz Biotechnology (Santa Cruz, CA, USA). All reagents and probes required to perform real-time PCR were from Thermo Fisher Scientific INC (Waltham, MA, USA). Specific polyclonal rabbit anti-mouse SPL antibodies were a kind gift from Dr R.L. Proia (National Institute of Diabetes and Digestive and Kidney Disease, Institute of Health, Bethesda, MD, USA).

### 2.2. Cell Culture

Murine C2C12 myoblasts were routinely grown in DMEM supplemented with 10% fetal bovine serum, 2 mM l-glutamine, 100 U/mL penicillin, and 100 μg/mL streptomycin at 37 °C in 5% CO_2_. To induce differentiation, confluent cells were cultured in DMEM supplemented with 2% HS for 4 days. For the experiments, myotubes were treated in DMEM without serum supplemented with 1 mg/mL BSA. When requested, cells were incubated with inhibitors 1 h before challenge with the agonist and/or inhibitors.

### 2.3. Quantitative Real-Time Reverse Transcription PCR

Total RNA from C2C12 myotubes was extracted using a TRI Reagent^®^ RNA Isolation Reagent. Then, 1–2 μg of RNA was reverse transcribed using the high capacity cDNA reverse transcription kit Thermo Fisher Scientific Inc. (Waltham, MA, USA). TaqMan gene expression assays were used to perform real-time PCR in order to quantify the mRNA expression of S1P metabolism enzymes (SK1, SK2, SPL, SPP1, SPP2), S1PR (S1P_1_, S1P_2_, S1P_3_, S1P_4_, S1P_5_) and S1P specific transporter Spns2. Each measurement was carried out in triplicate using the CFX96 Touch™ Real-Time PCR Detection System (Biorad, Hercules, CA, USA) as described previously [32,33], by simultaneous amplification of the target sequence together with the housekeeping gene β-actin. The 2^−ΔΔCt^ method was applied as a comparative method of quantification [34], and data were normalized to β-actin expression.

### 2.4. Western Blot Analysis

Myotubes were lysed for 20 min on ice in complete radio-immunoprecipitation assay (RIPA) buffer (150 mM NaCl, 100 mM NaF, 2 mM EGTA, 50 mM Tris HCl pH 7.5, 1 mM orthovanadate, 1% triton, 0.1% SDS, and 0.1% protease inhibitor cocktail). Lysates were clarified by centrifugation, and total protein contents were obtained by using Bradford assay (Bio-Rad Laboratories, Hercules, CA, USA) according to the manufacturer’s instructions. Samples resuspended in Laemmli’s SDS (sodium dodecyl sulphate) sample buffer were loaded on a 4–20% pre-cast-SDS-PAGE gels (Bio-Rad Laboratories, Hercules, CA, USA) and blotted onto PVDF membranes. Membranes were incubated overnight with the primary antibodies at 4 °C and then with horseradish peroxidase-conjugated secondary antibodies for 1 h at room temperature. Chemiluminescence was used to detect bound antibodies: immunoreactive bands were detected with an ECL kit detection system at Amersham Imager 600 (GE Healthcare, Chicago, IL, USA). For quantification, densitometric analysis of the bands was performed by using ImageJ software version 2.0.0-rc-64. The intensities of the immunostained bands were normalized on the expression of the housekeeping gene GAPDH from the same PVDF membrane.

### 2.5. Liquid Chromatography Tandem-Mass Spectrometry

Cell pellets or cell culture media were subjected to lipid extraction as described [35]. To this end, the extraction solvent, methanol/chloroform (2:1, *v/v*) for cell pellets and 1-butanol for media, contained the internal standards d_7_-dihydrosphingosine (d_7_-dhSph), d_7_-sphingosine (d_7_-Sph), d_7_-sphingosine 1-phosphate (d_7_-S1P) and C17Cer (d18:1/17:0) (all Avanti Polar Lipids, Alabaster, AL, USA). Final extracts were subjected to LC-MS/MS sphingolipid quantification applying the multiple reaction monitoring (MRM) approach. Chromatographic separation was achieved on a 1290 Infinity II HPLC (Agilent Technologies, Waldbronn, Germany) equipped with a Poroshell 120 EC-C8 column (3.0 × 150 mm, 2.7 μm; Agilent Technologies, Waldbronn, Germany) guarded by a pre-column (3.0 × 5 mm, 2.7 μm) of identical material. MS/MS analyses were carried out using a 6495 triple-quadrupole mass spectrometer (Agilent Technologies, Waldbronn, Germany) operating in the positive electrospray ionization mode (ESI+). Chromatographic conditions and settings of the ESI source and MS/MS detector have been published elsewhere [36]. The following mass transitions were recorded (qualifier product ions in parentheses): long-chain bases: *m/z* 300.3 → 282.3 (252.3) for Sph, *m/z* 302.3 → 284.3 (254.3) for dhSph, *m/z* 307.3 → 289.3 (259.3) for d_7_-Sph, *m/z* 309.4 → 291.3 (261.3) for d_7_-dhSph, *m/z* 380.3 → 264.3 (82.1) for S1P, and *m/z* 387.3 → 271.3 (82.1) for d_7_-S1P; ceramides (Cer): *m/z* 520.5 → 264.3 (282.3) for C16Cer, *m/z* 534.5 → 264.3 (282.3) for C17Cer, *m/z* 548.5 → 264.3 (282.3) for C18Cer, *m/z* 576.6 → 264.3 (282.3) for C20Cer, *m/z* 604.6 → 264.3 (282.3) for C22Cer, *m/z* 630.6 → 264.3 (282.3) for C24:1Cer, and *m/z* 632.6 → 264.3 (282.3) for C24Cer. Peak areas of Cer subspecies, as determined with MassHunter software (version 10.1, Agilent Technologies, Waldbronn, Germany), were normalized to those of the internal standard (C17Cer) followed by external calibration in the range of 1 fmol to 50 pmol on column. dhSph, Sph and S1P were directly quantified via their deuterated internal standards d_7_-dhSph (0.125 pmol on column), d_7_-Sph (0.25 pmol on column) and d_7_-S1P (0.125 pmol on column). Quantification was performed with MassHunter Software (Agilent Technologies, Waldbronn, Germany).

### 2.6. Electrophysiology

The electrophysiological features of C2C12 myotubes were analysed by the whole cell patch clamp technique under a Nikon Eclipse TE200 inverted microscope (Nikon Europe BV, 1076 ER Amsterdam, The Netherlands). Myotubes were plated on glass coverslips and cultured under the different conditions. The electrophysiological records were then achieved moving the coverslip with the adherent cells in the experimental chamber constantly superfused at a rate of 1.8 mL min^−1^ with physiological external solution (mM): 150 NaCl, 5 KCl, 2.5 CaCl_2_, 1 MgCl_2_, 10 D-glucose and 10 HEPES (pH 7.4 with NaOH). By using borosilicate glass capillaries (GC150-7.5; Clark, Electromedical Instruments, Reading, UK) we made the patch pipettes by means of a vertical puller (Narishige, Tokyo, Japan). Usually, the patch pipettes were filled with a standard internal solution having the following composition (mM): 130 KCl, 10 NaH_2_PO_4_, 0.2 CaCl_2_, 1 EGTA, 5 MgATP and 10 HEPES (pH 7.2 with KOH). Once filled, the pipette resistance was 3–7 MΩ. The apparatus for the electrophysiological measurements consisted of the Axopatch 200 B amplifier, A/D-D/A interfaces Digidata 1200; Pclamp 6 software (Axon Instruments, Foster City, CA, USA) as described previously [37,38]. In the current clamp mode of the 200 B amplifier, we recorded the resting membrane potential (RMP) by using a stimulus I = 0. In the voltage-clamp mode, we assessed the passive membrane properties by the application of a voltage pulse of ±10 mV starting from a holding potential (HP) = −70 mV. We analysed our records by the Clampfit 9 software (Axon Instruments, Foster City, CA, USA) and the decay of the recorded passive current was fitted by the sum of 2 exponential functions representing the time course of the surface and tubular membrane passive current [39,40]. The linear capacitance, C_m_, was estimated from the area beneath the capacitive transient current. Since the membrane-specific capacitance is assumed to be 1 μF/cm^2^, the C_m_ value can be used as an index of the cell surface area. The membrane resistance, R_m_, was calculated from the steady-state membrane current (I_m_) as described in previous paper [41]. To record the transmembrane ion currents, we used the voltage-clamp mode. In particular, the delayed rectifier K^+^ current (I_K_) was evoked by using a physiological external bath solution with Nifedipine (10 µM) to hamper the l-type Ca^2+^ current occurrence. In contrast, to record only Ca^2+^ current, I_Ca_, we used a Na^+^– and K^+^–free high-TEA external solution (mM): 10 CaCl_2_, 145 tetraethylammonium bromide, 10 HEPES and a suitable filling pipette solution (mM): 150 CsBr, 5 MgCl_2_, 10 ethylene-bis(oxyethylenenitrilo) tetraacetic acid (EGTA), 10 (4-(2-hydroxyethyl)-1-piperazineethanesulfonic acid) (HEPES) (pH = 7.2) [42]. A pulse protocol of stimulation consisting of 1-s step voltage pulses, ranging from −80 to 50 mV, in 10 mV increments was applied from a HP = −60 mV to elicit I_K_ or −80 mV to elicit I_Ca_. In any case, the capacitive and leak currents were removed on-line by the P4 procedure. To properly compare the currents evoked from myotubes of different dimension, we normalized the current amplitude values to C_m_. The ratio I/C_m_ (pA/pF) represents the current density. All drugs were from Merck Life Sciences (Burlington, MA, USA). The electrophysiological experiments were achieved at room temperature (about 22 °C).

### 2.7. Oxygen Consumption Assay

The Oxygen Consumption Rate (OCR) was measured by using a Clark-type O_2_ electrode inserted in a thermostated airtight chamber (Oxygraph Plus System, Hansatech Instruments) (King’s Lynn, Norfolk, UK). Control and treated myotubes (24 h) were trypsinized, washed with phosphate buffered saline and suspended in culture medium. Cell suspension was transferred to the airtight chamber maintained at 37 °C and the oxygen consumption was measured for 10 min. The OCR values, expressed as nmol of consumed O_2_/mL/min, were normalized to the cellular protein content as previously [43].

### 2.8. NMR-Based Metabolomic Analyses

Cell lysates and the respective conditioned media were analysed using an untargeted ^1^H NMR-based metabolomic approach [44]. Samples for NMR analyses were prepared according to procedures developed to obtain highly reproducible samples for cell metabolomics [45,46,47].

Cell lysates were obtained by sonication on ice and then centrifuged at 200,000× *g*, 1 h, 4 °C. Conditioned media were also collected and all the samples were stored at −80 °C.

Frozen samples were thawed in ice and shaken before use. NMR samples were prepared into 5.00 mm NMR tubes (Bruker BioSpin srl; Rheinstetten, Germany). For cell lysates, 50 μL of ^2^H_2_O were added to 450 μL of the lysate sample. In the case of cell culture media, an aliquot of 300 μL of sodium phosphate buffer (70 mM Na_2_HPO_4_; 20% *v*/*v* ^2^H_2_O; 4.6 mM TMSP, pH 7.4) was added to 300 μL of each medium sample. The mixtures were homogenized by vortexing for 30 s and transferred into 5 mm NMR tubes for analysis.

NMR spectral acquisition and processing were performed according to optimized procedures for metabolomic analysis of cell lysates and media developed at CERM [44,48].

One-dimensional (1d) ^1^H NMR spectra were acquired on each sample using a Bruker 600 MHz spectrometer (Bruker BioSpin), operating at 600.13 MHz proton Larmor frequency and equipped with a 5 mm PATXI ^1^H-^13^C-^15^N and ^2^H-decoupling probe including a z-axis gradient coil, an automatic tuning-matching (ATM) and an automatic and refrigerate sample changer (SampleJet, Bruker BioSpin srl; Rheinstetten, Germany). A BTO 2000 thermocouple served for temperature stabilization at the level of approximately 0.1 K of the sample. ^1^H NMR spectra were acquired at 300 K with water presaturation and a 1d nuclear Overhauser enhancement spectroscopy (NOESY) pulse sequence (noesygppr1d, Bruker). A total of 256 and 64 scans, for lysates and conditioned media, respectively, were used, with 98,304 data points, a spectral width of 18,028 Hz, an acquisition time of 2.7 s, a relaxation delay of 4 s and a mixing time of 0.1 s.

The raw data were multiplied by a 0.3 Hz exponential line broadening and Fourier transformation were applied. Transformed spectra were automatically corrected for phase and baseline distortions. The calibration of the spectra was performed to the signal of alanine at 1.49 ppm (^1^H chemical shift).

The most abundant metabolites present in the spectra were assigned and their levels analysed. The assignment procedure was made up using an NMR spectra library of pure organic compounds, stored reference NMR spectra of metabolites and literature data. Matching between new NMR data and databases was performed using the AssureNMR software (Bruker, BioSpin srl; Rheinstetten, Germany). The relative concentrations of the various metabolites were calculated by integrating the corresponding signals in the spectra using a home-made R script. The non-parametric Wilcoxon test was used for the determination of the meaningful metabolites: a *p*-value of 0.05 was considered statistically significant.

### 2.9. Statistical Analysis

To perform densitometric analysis of the Western blot bands and graphical representations, ImageJ software and GraphPad Prism 6.0 (GraphPad Software, San Diego, CA, USA) were utilized, respectively. Statistical analysis was performed using Student’s *t*-test, one-way and two-way ANOVA followed by Bonferroni’s or Tukey’s post hoc test and paired Wilcoxon test.

## 3. Results

### 3.1. Adiponectin Modulates S1P Signalling in Myotubes

**Figure 1:**
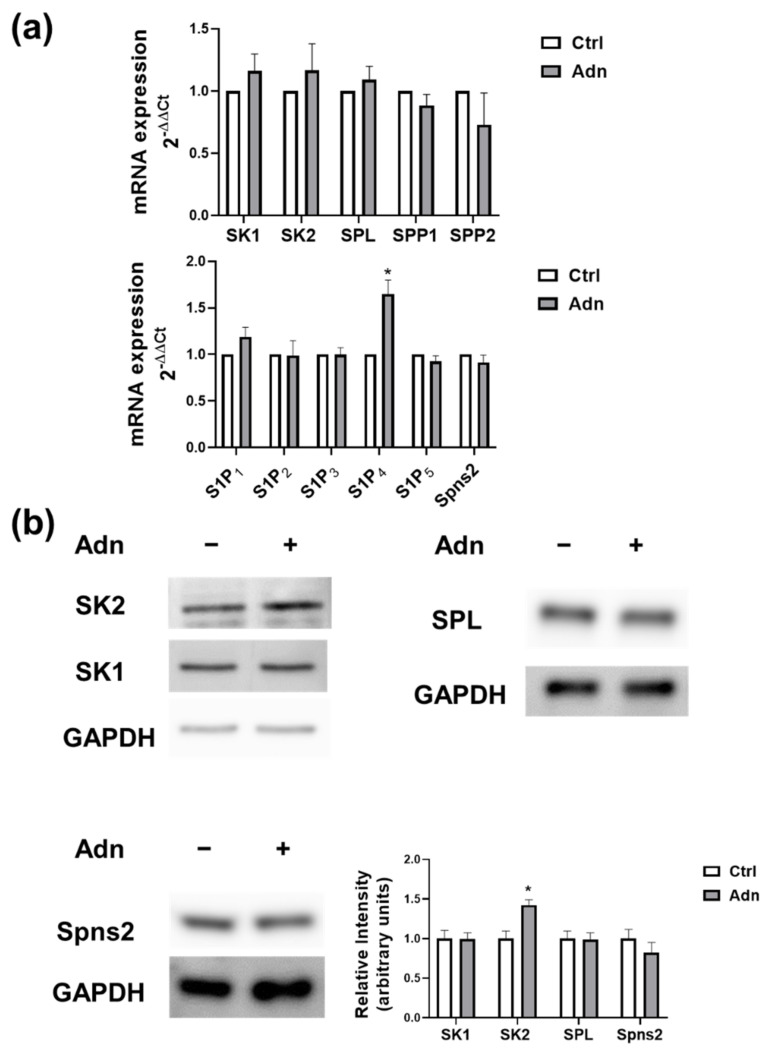
Effect of Adiponectin on S1P metabolism and signalling. (**a**) Quantitative mRNA analysis was performed by real-time PCR in total RNA extracted from C2C12 myotubes stimulated or not with 1 µg/mL Adn for 15 h. mRNA quantitation of S1P metabolism enzymes (SK1, SK2, SPL, SPP1 and SPP2), S1PR (S1P_1_, S1P_2_, S1P_3_, S1P_4_ and S1P_5_) and S1P specific transporter Spns2 was based on the 2^−ΔΔCt^ method, using individual enzyme/receptor/S1P transporter of the unchallenged specimen as calibrator. Data are the mean ± SEM of three independent experiments, each performed in triplicate. (**b**) C2C12 myotubes were incubated for 24 h in the absence or in the presence of 1 µg/mL Adn. Aliquots of total cell lysates were used to perform Western analysis, using specific anti-SK1, anti-SK2, anti-SPL and anti-Spns2 antibodies. A representative blot is shown. The histogram represents the densitometric analysis of at least three independent experiments, each performed in triplicate. Data are the mean ± SEM and are reported as protein expression normalized to GAPDH, fold change over control (set as 1). The effect of Adn on S1P_4_ mRNA and SK2 protein levels was statistically significant by Student’s *t*-test (* *p* < 0.05).

Firstly, we examined whether Adn was capable of regulating metabolism and signalling of S1P in C2C12 myotubes. Real Time PCR data, illustrated in Figure 1a (upper panel), showed that the treatment with 1 µg/mL globular Adn for 15 h did not significantly alter the expression of enzymes involved in S1P metabolism.

**Figure 2:**
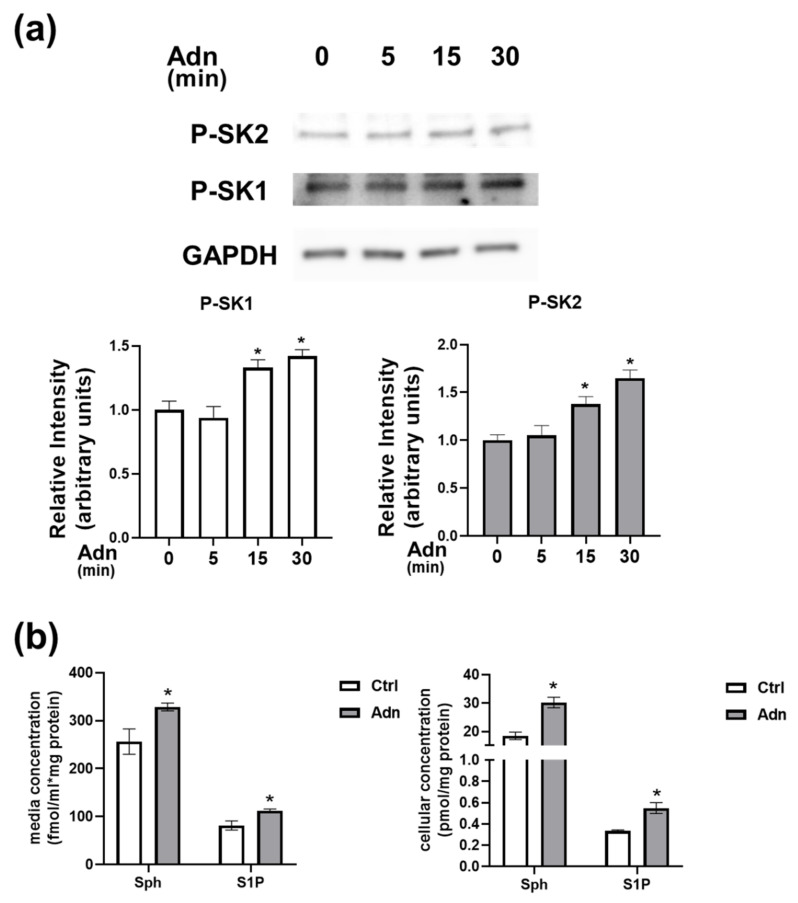
Adiponectin induces S1P biosynthesis. (**a**) C2C12 myotubes were incubated for the indicated time intervals in the absence or in the presence of 1 µg/mL Adn. Western blot analysis was performed using specific anti-phospho-SK1 and anti-phospho-SK2 antibodies. A blot representative of at least three independent experiments with analogous results is shown. The histogram represents densitometric analysis of three independent experiments each performed in triplicate. Data are the mean ± SEM and are reported as protein expression normalized to GAPDH, fold change over control (set as 1). The increase in phospho-SK1 and phospho-SK2 content induced by Adn was found to be statistically significant by one-way ANOVA followed by Bonferroni’s post hoc test (* *p* < 0.05, treated vs. control). (**b**) C2C12 myotubes were incubated for 1 h in the absence or in the presence of 1 µg/mL Adn before the media were collected (left panel) and cells harvested (right panel) and then subjected to Sph and S1P analysis. Results are reported as mean ± SEM of fmol/mL (media) or pmol (cell lysates) of S1P and Sph normalized on protein content. The increase of S1P and Sph levels induced by Adn was statistically significant by Student’s *t*-test * *p* < 0.05, treated vs Ctrl.

Treatment with globular Adn significantly increased the expression of S1P_4_ while the mRNA content of the other S1PR or the specific S1P transporter Spns2 (Figure 1a, lower panel) were unaffected by the treatment. No significant effects were observed when real time PCR analysis was performed after 6 and 24 h challenge with the hormone (data not shown). We then investigated whether Adn was able to regulate the protein content of S1P metabolism enzymes. Western blot analysis showed that the stimulation with 1 µg/mL Adn for 24 h augmented the protein content of SK2 while it did not alter SK1 protein levels in myotubes (Figure 1b). Moreover, Adn did not modulate the protein content of SPL and Spns2 (Figure 1b). Since agonist-induced stimulation of SK1/SK2 activity and translocation to the plasma membrane is dependent on enzyme phosphorylation [49,50], Western blot analysis using specific anti-phospho-SK1 or anti-phospho-SK2 antibodies was performed. Data reported in Figure 2a showed that cell challenge with the hormone (1 µg/mL) increased phosphorylation of SK1 and SK2, detectable in both cases at 15 min of incubation and persistent after 30 min of treatment. Collectively, these data indicate that Adn induces a rapid and persistent activation of SK1 and SK2 in myotubes, accompanied by the late augmented SK2 protein expression. In accordance with the rapid activation of SK1 and SK2, 1 h treatment with 1 µg/mL Adn significantly enhanced S1P in C2C12 myotubes (Figure 2b). In Figure 2 it is shown that also sphingosine levels were increased by Adn challenge, possibly due to the ceramidase activity of AdipoRs. In this regard, in myotubes pre-treated with the ceramidase inhibitor d-erythro-MAPP (5 µM) 45 min before agonist treatment, the reduction of Cer levels induced by the hormone was no more evident (Figure S1).

### 3.2. Role of S1P Biosynthesis in the Effect of Adn on Myotube Electrophysiological Properties

**Figure 3:**
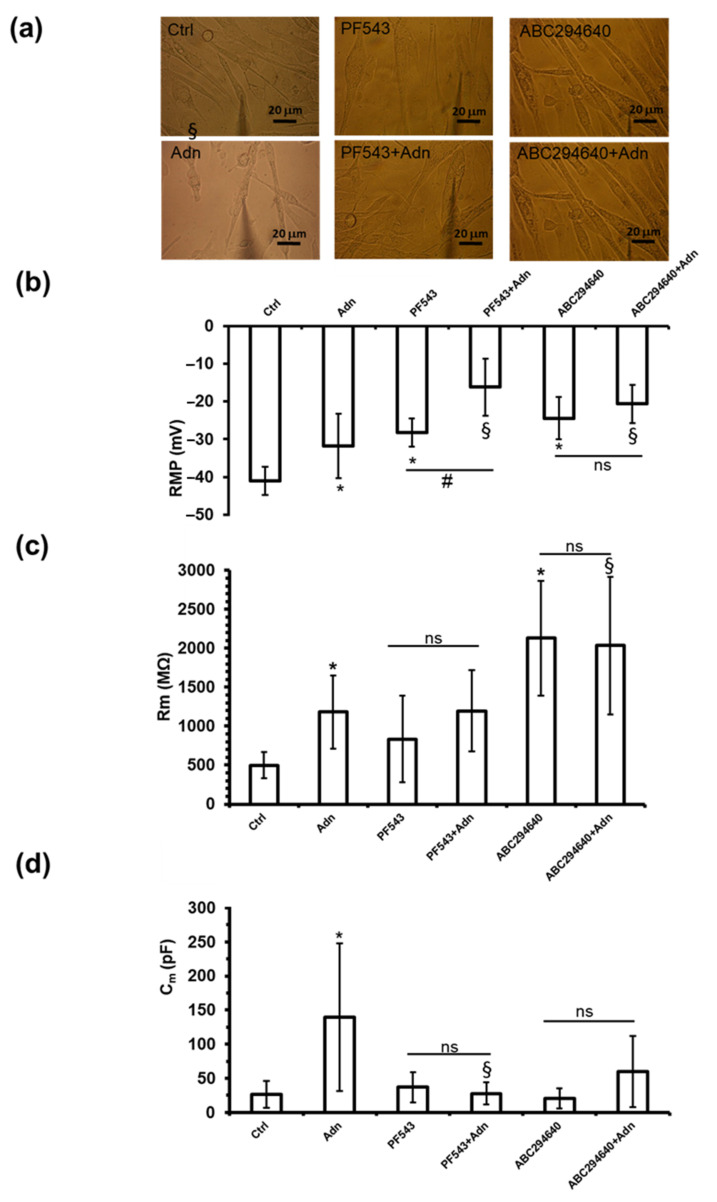
Role of S1P biosynthesis in the effect of Adn on the passive electrophysiological properties of C2C12 myotubes. (**a**) Representative photographs of Ctrl patched myotubes and of Adn, PF543, PF543 + Adn, ABC294640 and ABC294640 + Adn treated myotubes (Magnification 40×; scale bar 20 µm). C2C12 myotubes were pre-treated with specific SK1 inhibitor PF543 (10 µM) or SK2 inhibitor ABC294640 (1 µM) for 1 h before being challenged with 1 µg/mL Adn for 24 h. (**b**) Resting membrane potential (RMP) evaluated in current-clamp mode (Ctrl, *n* = 8; Adn, *n* = 21; PF543, *n* = 6; PF543 + Adn, *n* = 10; ABC294640 *n* = 7; ABC294640 + Adn, *n* = 8). (**c**) Membrane resistance (Rm) (Ctrl, *n* = 19; Adn, *n* = 39; PF543, *n* = 16; PF543 + Adn, *n* = 13; ABC294640, *n* = 10; ABC294640 + Adn, *n* = 14). (**d**) Membrane capacitance (C_m_) estimated in voltage-clamp (Ctrl, *n* = 8; Adn, *n* = 29; PF543, *n* = 12; PF543 + Adn, *n* = 19; ABC294640, *n* = 6; ABC294640 + Adn, *n* = 8). In all the panels values are means ± SD. Data are from three independent experiments, each performed in duplicate, *n* is the total number of analyzed myotubes. One-way ANOVA with Bonferroni’s post hoc test, * indicates *p* < 0.05 vs. Ctrl; # *p* < 0.05 vs. PF453; ns, not significant. The pharmacological blockade of SK1 (10 µM PF543) or SK2 (1 μM ABC294640) affects Adn-induced changes in electrophysiological passive properties in a statistically significant way by two-way ANOVA followed by Bonferroni’s post hoc test, § *p* < 0.05.

We investigated the effect of Adn on myotube membrane phenomena related to the excitability and excitation-contraction coupling by whole cell patch clamp analysis (Figure 3a). Moreover, in order to elucidate the possible involvement of S1P signalling on Adn effect, myotubes were pre-treated with the selective inhibitor of SK1, PF543 (10 µM), or of SK2, ABC294640 (1 µM), before being challenged with Adn 1 µg/mL for 24 h. Adn treatment significantly affected the resting membrane potential (RMP) compared to control, causing a statistically significant depolarization (Figure 3b). The treatment with PF543 and ABC294640 alone significantly depolarized the RMP compared to Ctrl. The Adn-induced RMP values measured in the presence of SK1 or SK2 inhibitors were statistically different to those observed without the inhibitors. While Adn continued to exert its action in membrane depolarization in the presence of SK1 inhibitor, when SK2 was blocked by ABC294640, Adn was unable to further depolarize the membrane.

The treatment of myotubes with Adn 1 µg/mL for 24 h significantly increased the membrane resistance (R_m_) compared to Ctrl (Figure 3c), thus causing a reduction of membrane permeability. The treatment with SK1 inhibitor did not induce significant alterations of the R_m_, while a different outcome was observed with ABC294640 treatment that significantly increased Rm value compared to Ctrl myotubes, suggesting a role of SK2 isoform in the maintenance of a proper membrane resistance. Interestingly, in the presence of SK2 inhibitor (1 µM ABC294640), but not that of SK1 (10 µM PF543), the increase of the Rm value induced by Adn was blocked, demonstrating a role for SK2 in the observed biological effect (Figure 3c).

Moreover, Adn treatment significantly increased the membrane capacitance (C_m_), assumed as index of surface membrane in C2C12 myotubes (Figure 3d). None of the inhibitors, given alone, altered per se the C_m_ value. Adn given in the presence of PF543, but not ABC294640, was no more able to increase the Cm value, highlighting a crucial role for SK1 in the effect of Adn (Figure 3d) on inducing C_m_ increase in myotubes.

**Figure 4:**
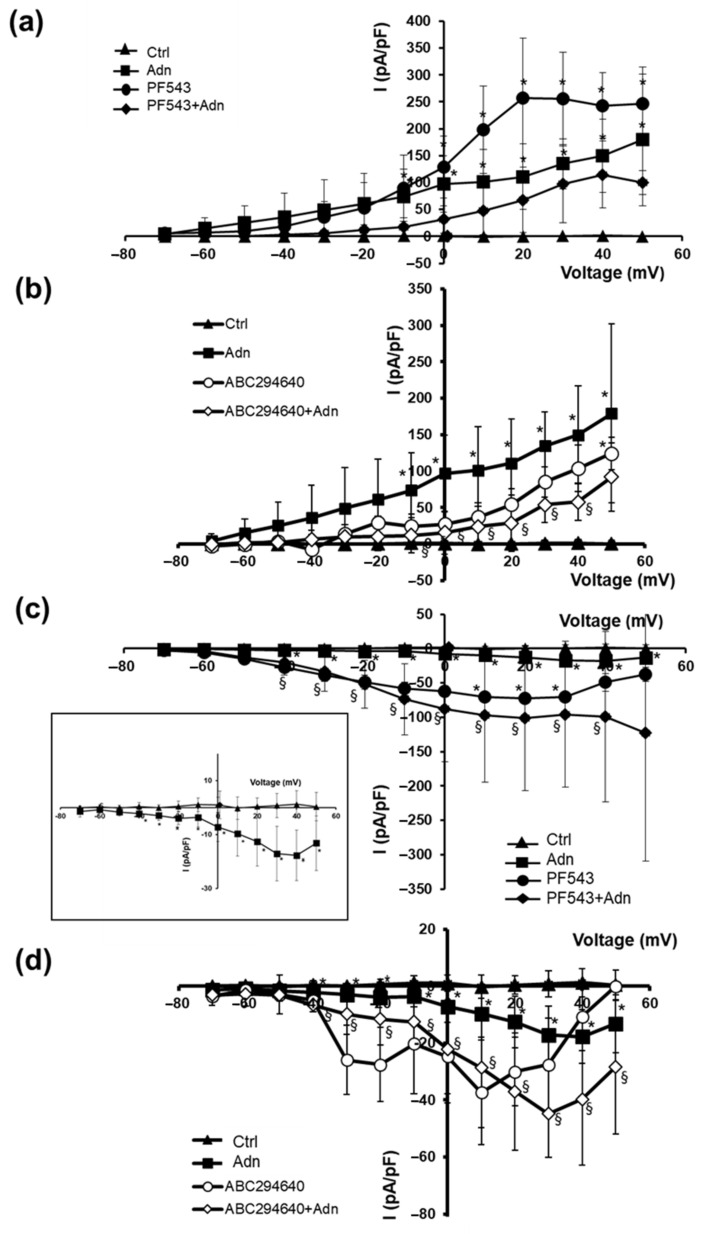
Role of S1P biosynthesis in the effect of Adn on ionic currents in C2C12 myotubes. C2C12 myotubes were pre-treated with specific SK1 inhibitor PF543 (10 µM) or SK2 inhibitor ABC294640 (1 µM) for 1 h before being challenged with 1 µg/mL Adn for 24 h. Overall I–V plots related to K^+^ currents measured in the presence of PF543 (**a**) or ABC294640 (**b**) are shown: Ctrl, filled triangles, *n* = 7; Adn, filled squares, *n* = 7; PF543, filled circles, *n* = 5; PF543 + Adn, filled diamonds, *n* = 3; ABC294640, open circles, *n* = 3; ABC294640 + Adn, open diamonds, *n* = 4). Adn increases K^+^ current compared to Ctrl (**a**,**b**), * *p* < 0.05 vs. Ctrl, § *p* < 0.05 vs. Adn (two-way ANOVA and Bonferroni’s post hoc test). Overall I–V plots related to Ca^2+^ currents measured following the pre-treatment with SK1 inhibitor PF543 10 µM (**c**) or SK2 inhibitor ABC294640 (1 µM) (**d**): Ctrl, filled triangles, *n* = 7; Adn, filled squares, *n* = 5; PF543, filled circles, *n* = 2; PF543 + Adn, filled diamonds, *n* = 4; ABC294640, open circles, *n* = 3; ABC294640 + Adn, open diamonds, *n* = 3). Adn increases Ca^2+^ inflow compared to Ctrl cells (**c**,**d**), * *p* < 0.05 vs. Ctrl, § *p* < 0.05 vs. Adn (two-way ANOVA and Bonferroni’s post hoc test). Data points are mean ± SD. Data are from three independent experiments, each performed in duplicate, *n* is the total number of analyzed myotubes. Error bars are shown when their size is bigger than the symbol.

In addition, we tested the effect of Adn on myotube ion currents (Figure 4) supposed to be crucial in the control of the excitability and Ca^2+^ entry in muscle cells. Treatment of myotubes with 1 µg/mL Adn for 24 h caused an increase of outward K^+^ currents compared to Ctrl, that was statistically significant from voltages more positive than −10 mV (Figure 4a,b, filled squares). In contrast, when added in the concomitant presence of SK2 inhibitor ABC294640 (ABC294640 + Adn, Figure 4b) but not SK1 inhibitor PF543 (PF543 + Adn, Figure 4a), Adn effect on I_K_ amplitude was hampered starting from −10 mV voltage. Of note, in the presence of PF543 the outward currents significantly increased compared to Ctrl, suggesting a role of SK1 per se in the modulation of the appropriate ion channel permeability. Overall, data related to the potentiating effects of Adn on K^+^ currents on myotubes (Figure 4a,b) suggest a role of SK2 activation in the final outcome of the hormone.

Finally, the effect of Adn on inward Ca^2+^ currents was investigated (Figure 4c,d, filled squares). Although the ionic currents showed a very small amplitude in control myotubes, the challenge with 1 µg/mL Adn for 24 h, caused a significant increase of inward current starting from −40 mV (Figure 4c, inset). When S1P biosynthesis was pharmacologically inhibited by blocking SK1 with 10 µM PF543 (PF543 + Adn, Figure 4c) and SK2 with 1 µM ABC294640 (ABC294640 + Adn, Figure 4d),the Adn-induced increase of inward Ca^2+^ currents was altered, suggesting a role of SK1 and SK2 in Adn effect. Lastly, while the treatment of C2C12 myotubes with ABC294640 alone did not cause a significant increase of the current amplitude compared to Ctrl treatment of myotubes, with PF543 alone increased the current amplitude compared to Ctrl, showing significant differences for various voltage pulses.

### 3.3. Role of S1P Biosynthesis in the Effect of Adn on Mitochondrial Function in Myotubes

To provide information about the influence of S1P metabolism on the Adn positive effects towards the mitochondrial oxidative phosphorylation, we examined myotube oxygen consumption rate (OCR) using a Clark-type electrode inserted into a thermostated airtight chamber, as described [43].

**Figure 5:**
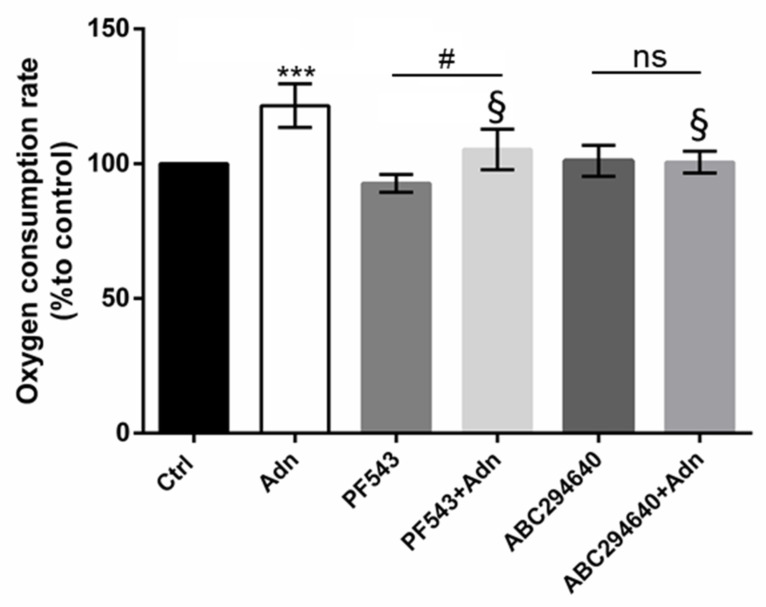
Role of S1P biosynthesis in the oxidative metabolism of Adn in C2C12 myotubes. C2C12 myotubes were pre-treated with specific SK1 inhibitor PF543 (10 µM) or SK2 inhibitor ABC294640 (1 µM) for 1 h before being challenged with 1 µg/mL Adn for 24 h. The percentage of oxygen consumption rate (OCR) values were measured in cell lysates using a Clark-type O_2_ electrode from Hansatech Instruments as described in Section 2. Histogram reports the percentages of mean values ± SD with respect to controls. Data are obtained from at least three independent experiments, each performed in duplicate. The statistical analysis was carried out using one-way ANOVA test followed by Tukey’s multiple comparisons test using GraphPad Prism software v 6.0 (*** *p* < 0.001 vs. Ctrl; # *p* < 0.05 vs. PF543, § *p* < 0.01 vs. Adn, ns, not significant).

The results confirm the well-known ability of Adn to increase cellular respiration (Figure 5). Figure 5 shows that when SK1 is inhibited pre-treating with PF543 (10 µM) Adn is still able to increase OCR, even if to a minor extent compared to control condition. However, SK2 inhibition by ABC294640 (1 µM) totally prevented OCR modulation by Adn, demonstrating that S1P synthesis, mainly by SK2, is crucially involved in the modulation of myotube OCR by Adn.

### 3.4. Role of S1P Biosynthesis in the Metabolome of Adn-Treated C2C12 Myotubes

**Figure 6:**
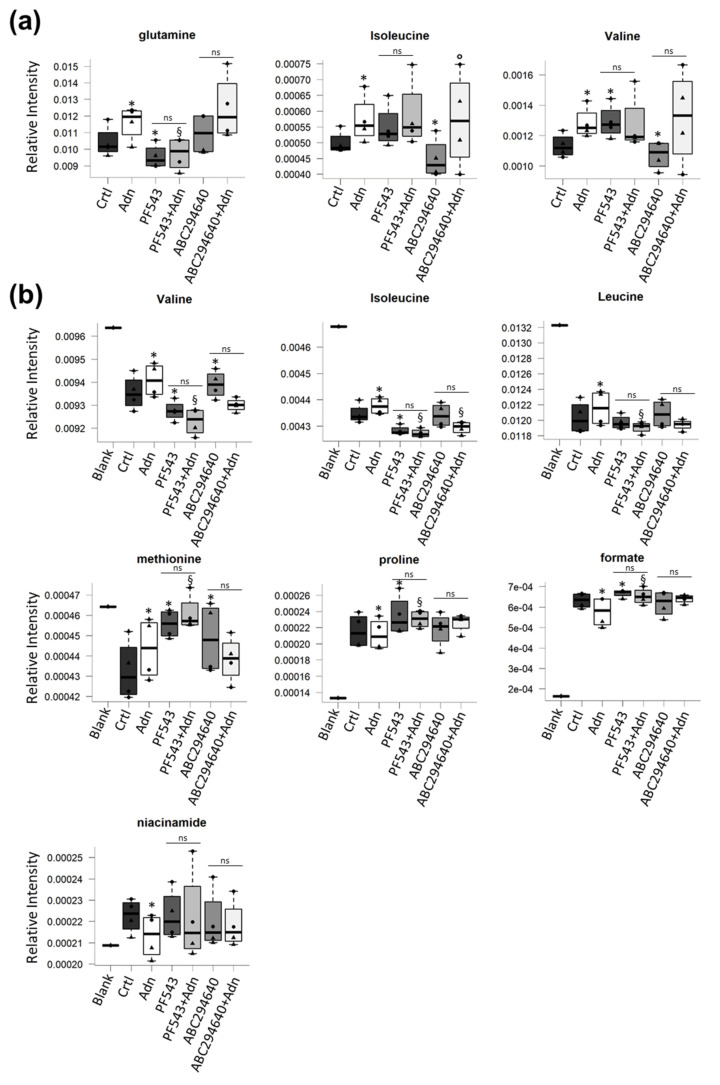
Role of S1P biosynthesis in the metabolome of Adn-treated C2C12 myotubes. C2C12 myotubes were pre-treated with specific SK1 inhibitor PF543 (10 µM) or SK2 inhibitor ABC294640 (1 µM) for 1 h before being challenged with 1 µg/mL Adn for 24 h. Adn-dependent metabolomic changes in cell lysates (endo-metabolome) (**a**) and conditioned media (exo-metabolome) (**b**) were analysed in four experiments. Boxplots of the significant metabolites: changes in metabolite levels caused by Adn were statistically significant by paired Wilcoxon test, (* *p* < 0.05 vs. Ctrl, § *p* < 0.05 vs. Adn, ° *p* < 0.05 vs. ABC294640, ns, not significant).

The metabolomic profile of C2C12 myotubes treated with Adn for 24 h was characterized acquiring ^1^H NMR spectra using a 600 MHz spectrometer optimized for metabolomics analysis. From the overall analysis of the Adn-dependent changes in the cell metabolome (Figure 6, Figures S2 and S3), of particular interest is the significant increase of the intracellular levels of branched amino acids (valine and isoleucine) and glutamine (Figure 6a). Interestingly when SK1 activation was inhibited by PF543 (10 µM), glutamine increase induced by Adn on myotubes was blocked. On the contrary, SK2 inhibition did not altered Adn-induced increase of intracellular amino acid levels (Figure 6a).

The analysis of the extracellular metabolomic profiles (exo-metabolome) indicated that the uptake of branched amino acids from the media was significantly reduced following Adn treatment (Figure 6b). Furthermore, the exo-metabolome analysis highlighted a reduced uptake of methionine in addition to a reduced release of proline, formate and niacinamide in myotubes treated with Adn compared to control (Figure 6b). When myotubes were pre-treated with SK1 inhibitor PF543, the Adn-induced metabolic changes in the exometabolome were no more significant with the exception of niacinamide, suggesting the requirement for SK1 activation for these Adn-induced effects. Moreover, the Adn-induced decreased uptake of isoleucine was also found to depend on SK2 activation (Figure 6b).

## 4. Discussion

Skeletal muscle is necessary for many fundamental biological processes in the human body such as movement, posture and energy homeostasis. A tight balance between protein synthesis and degradation is required to maintain muscle homeostasis [51].

Consolidated literature data support a crucial role of the bioactive sphingolipid S1P in the biology of skeletal muscle [26,52,53,54,55]. S1P indeed plays a crucial role in satellite cell activation [27], proliferation [4], myoblast differentiation [28] and migration [56]. More importantly, S1P signalling axis is emerged to be under the control of numerous well-known physiological and pathological cues that regulate fundamental skeletal muscle processes such as PDGF [53], IGF-1 [52] TGFβ [57,58] and TNFα [37,54].

Adn is a crucial hormone endowed with insulin-sensitizing and anti-inflammatory properties [13]. Adn is released by adipocytes but also by other cell types such as skeletal muscle cells [15]. In addition to its effects on energy homeostasis, Adn has been shown to exert a positive action on the differentiation of myoblasts [13].

Recently, several studies have shown that different biological effects of Adn depend on its ability of decreasing ceramide levels and augmenting those of S1P [22,59,60], although the functional cross-talk between the hormone and the bioactive sphingolipid signalling axis in skeletal muscle is not fully elucidated.

Ceramide and S1P are bioactive molecules that regulate numerous and opposite cellular functions: while ceramide exerts anti-proliferative and apoptotic actions [61,62], S1P is reported to stimulate cell proliferation and survival pathways [62]. The control of cell fate by these two interconvertible sphingolipids has been nominated “the sphingolipid rheostat” and SK plays a crucial role in the regulation of the dynamic ratio between the two molecules which constitutes a physiological cellular sensor [61].

In the present manuscript, we provide the first experimental evidence that S1P signalling axis is modulated by globular Adn in C2C12 murine myotubes since hormone challenge resulted not only in the increase of expression levels of SK2 and S1P_4_, but also in the activation of both SK1 and SK2, as evidenced by their increased phosphorylation. These latter findings were confirmed by the observed increase of the S1P content, quantified by LC-MS/MS analysis, after Adn treatment. These findings strengthen the hypothesis that Adn potently modulates sphingolipid levels in skeletal muscle cells, in agreement with previous reports [23]. It has been shown that ceramidase activity associated with AdipoRs represents a key molecular mechanism by which the broad spectrum of effects are elicited by the hormone in hepatocytes, cardiomyocytes and pancreatic β-cells [15]. Recent reports have indeed suggested that in the “progesterone and Adn Q receptor (PAQR) family” of proteins the ceramidase activity may be an integral component of the receptors themselves [63]. Accordingly, we observed that pre-treatment of myotubes with the ceramidase inhibitor D-erythro-MAPP, impedes the reduction of ceramides provoked by Adn.

From a functional point of view, here, we demonstrated for the first time that S1P biosynthesis is necessary for the Adn ability to positively influence electrophysiological properties as well as oxidative metabolism in C2C12 myotubes, these effects being inhibited when SK1 and/or SK2 were pharmacologically blocked. The observation that both SK isoforms have been here demonstrated to be required for Adn-induced biological effects in myotubes is not surprising since, although SK1 and SK2 might display different cellular functions (such as on cell survival and proliferation) due to different enzymatic activity regulation and diverse cellular localization [64], some redundancy between these two enzyme isoforms has been demonstrated, for example, in IGF-1-induced myoblast differentiation [52] and vessel formation by endothelial and mesoangioblast cells [65]. Moreover, mice deficient in either SK1 or SK2 had no obvious abnormalities, whereas double-knockout animals were embryonic lethal [66].

SK inhibitors have been extensively employed to demonstrate the involvement of S1P axis in Adn-induced actions [15,60,67]. However, it remains to be determined whether the levels of other bioactive sphingolipids sphingosine and ceramides change following SK inhibitor treatment, taking into consideration the crucial role of the sphingolipid rheostat in determining cell fate.

In this work, we showed that Adn challenge depolarized the resting membrane potential, bringing it closer to the threshold for the onset of the action potential, thus enhancing its excitability. Moreover, Adn augmented R_m_ improving the non-selective control of membrane permeability, contributing with a protective/repairing effect that can be particularly favourable in injured cell [68,69]. Lastly, it augmented the membrane capacitance, suggesting a further positive effect on myotube enlargement and/or a possible increase of t-tubular system. In fact, the C_m_ parameter is assumed to reflect a quantitative relationship among cell length, width, volume (taking into account the contribution of membrane infoldings), tubules and caveolae [70].

Interestingly, Adn increased the ion current size. This effect was particularly marked for outward currents at positive potentials starting from −10 mV and may suggest the Adn ability to facilitate a faster return to resting potentials after depolarizing stimuli, such as after an action potential. With regard to Ca^2+^ currents, the increase in amplitude evoked by Adn was hampered when S1P biosynthesis was prevented. Notably, the blockade of SK1 altered per se Ca^2+^ and K^+^ currents in the absence of Adn as previously reported [37], supporting the hypothesis that, in control conditions, SK1 activity and the consequent S1P formation may have a role in the modulation of the appropriate ion channel permeability and may help to prevent an excessive Ca^2+^ entry through voltage dependent Ca^2+^ channels. With regard to SK2 inhibitor treatment alone, this induced the appearance of highly variable currents, but not significantly different to those recorded in control condition, suggesting that the enzyme is not involved per se in the control of ion channel opening.

In line with data reported in literature [71], Adn significantly stimulated oxygen consumption. In the presence of SK2 pharmacological inhibitor, the increased oxygen consumption induced by Adn was no more evident.

Remarkably, NMR-based metabolomics analysis demonstrated that Adn-induced increase of glutamine depended on S1P biosynthesis. Given the well-known role of Adn in the activation of adenosine monophosphate-activated protein kinase (AMPK) [71,72] and AMPK inhibition on the mammalian target of rapamycin (mTOR) [73], it should be hypothesized that Adn stimulates aminoacid employment as energy substrate further supporting the evidence of the augmented oxygen consumption by the hormone.

Previous findings support a role of globular Adn [69,74,75,76], as well as of the small molecule AdipoR agonist, GTDF [77], in efficaciously protecting against skeletal muscle atrophy induced by various catabolic stimuli both in vitro and in vivo. In contrast, AdipoRon treatment has been shown to reduce the content of skeletal muscle proteins and myotube size in C2C12 cells, and diminished muscle fibre size in mouse plantaris muscle [78] and Adn levels were found to be significantly elevated in sarcopenic males and negatively correlated with functional measures [79]. Further studies are required to resolve these apparently contrasting roles of Adn in the regulation of muscle mass.

S1P has previously been demonstrated to activate AMPK [80] and numerous findings from the literature demonstrates that S1P increases calcium influx [81] and Ca^2+^ release from the endoplasmic reticulum [80]. Since AMPK is a well-known effector of Adn in different cell types and the regulation of this kinase by S1P is recognized, a future systematic study devoted to dissecting the underlying molecular mechanisms is required to clarify whether S1P signalling is upstream or downstream Adn-induced AMPK activation and if S1P metabolism regulates its activation.

Interestingly, in conditioned media, the Adn-induced decrease of the levels of formate depended on S1P metabolism, being the effect blocked in the presence of SK1 inhibitor. The reduced release of niacinamide and formate by Adn might support a role for the hormone in NAD^+^ and nucleotide synthesis, respectively [82,83].

Altogether, the findings reported here clearly show that S1P metabolism is a key mediator of the electrophysiological properties and oxidative metabolism of Adn in C2C12 myotubes, further elucidating the molecular mechanism by which this hormone exerts its positive effects in skeletal muscle.

## Data Availability

Data are contained within the article or Supplementary Materials.

## Supplementary Materials

The following supporting information can be downloaded at: https://www.mdpi.com/article/10.3390/cells11040713/s1, Figure S1: Adn-induced reduction of Cer levels depends on ceramidase activity. Figure S2: ^1^H-NMR-based endometabolome profiles. Figure S3: ^1^H-NMR-based exometabolome profiles.

## References

[1] Bonaldo P., Sandri M. (2013). Cellular and Molecular Mechanisms of Muscle Atrophy. Dis. Model. Mech..

[2] Phielix E., Mensink M. (2008). Type 2 Diabetes Mellitus and Skeletal Muscle Metabolic Function. Physiol. Behav..

[3] Erekat N.S. (2015). Apoptotic Mediators Are Upregulated in the Skeletal Muscle of Chronic/Progressive Mouse Model of Parkinson’s Disease. Anat. Rec..

[4] Calise S., Blescia S., Cencetti F., Bernacchioni C., Donati C., Bruni P. (2012). Sphingosine 1-Phosphate Stimulates Proliferation and Migration of Satellite Cells: Role of S1P Receptors. Biochim. Biophys. Acta BBA Mol. Cell Res..

[5] Hotta K., Funahashi T., Arita Y., Takahashi M., Matsuda M., Okamoto Y., Iwahashi H., Kuriyama H., Ouchi N., Maeda K. (2000). Plasma Concentrations of a Novel, Adipose-Specific Protein, Adiponectin, in Type 2 Diabetic Patients. Arter. Thromb. Vasc. Biol..

[6] Scherer P.E., Williams S., Fogliano M., Baldini G., Lodish H.F. (1995). A Novel Serum Protein Similar to C1q, Produced Exclusively in Adipocytes. J. Biol. Chem..

[7] Fiaschi T., Magherini F., Gamberi T., Modesti P.A., Modesti A. (2014). Adiponectin as a Tissue Regenerating Hormone: More than a Metabolic Function. Cell. Mol. Life Sci..

[8] Waki H., Yamauchi T., Kamon J., Kita S., Ito Y., Hada Y., Uchida S., Tsuchida A., Takekawa S., Kadowaki T. (2005). Generation of Globular Fragment of Adiponectin by Leukocyte Elastase Secreted by Monocytic Cell Line THP-1. Endocrinology.

[9] Yamauchi T., Kamon J., Ito Y., Tsuchida A., Yokomizo T., Kita S., Sugiyama T., Miyagishi M., Hara K., Tsunoda M. (2003). Cloning of Adiponectin Receptors That Mediate Antidiabetic Metabolic Effects. Nature.

[10] Tishinsky J.M., Robinson L.E., Dyck D.J. (2012). Insulin-Sensitizing Properties of Adiponectin. Biochimie.

[11] Kadowaki T., Yamauchi T., Kubota N., Hara K., Ueki K., Tobe K. (2006). Adiponectin and Adiponectin Receptors in Insulin Resistance, Diabetes, and the Metabolic Syndrome. J. Clin. Investig..

[12] Yamauchi T., Kamon J., Waki H., Terauchi Y., Kubota N., Hara K., Mori Y., Ide T., Murakami K., Tsuboyama-Kasaoka N. (2001). The Fat-Derived Hormone Adiponectin Reverses Insulin Resistance Associated with Both Lipoatrophy and Obesity. Nat. Med..

[13] Fiaschi T., Cirelli D., Comito G., Gelmini S., Ramponi G., Serio M., Chiarugi P. (2009). Globular Adiponectin Induces Differentiation and Fusion of Skeletal Muscle Cells. Cell Res..

[14] Idrizaj E., Garella R., Nistri S., Dell’Accio A., Cassioli E., Rossi E., Castellini G., Ricca V., Squecco R., Baccari M.C. (2020). Adiponectin Exerts Peripheral Inhibitory Effects on the Mouse Gastric Smooth Muscle through the AMPK Pathway. Int. J. Mol. Sci..

[15] Holland W.L., Miller R.A., Wang Z.V., Sun K., Barth B.M., Bui H.H., Davis K.E., Bikman B.T., Halberg N., Rutkowski J.M. (2011). Receptor-Mediated Activation of Ceramidase Activity Initiates the Pleiotropic Actions of Adiponectin. Nat. Med..

[16] Maceyka M., Harikumar K.B., Milstien S., Spiegel S. (2012). Sphingosine-1-Phosphate Signaling and Its Role in Disease. Trends Cell Biol..

[17] Adams D.R., Pyne S., Pyne N.J. (2020). Structure-Function Analysis of Lipid Substrates and Inhibitors of Sphingosine Kinases. Cell. Signal..

[18] Blaho V.A., Hla T. (2014). An Update on the Biology of Sphingosine 1-Phosphate Receptors. J. Lipid. Res..

[19] Kawahara A., Nishi T., Hisano Y., Fukui H., Yamaguchi A., Mochizuki N. (2009). The Sphingolipid Transporter Spns2 Functions in Migration of Zebrafish Myocardial Precursors. Science.

[20] Vu T.M., Ishizu A.-N., Foo J.C., Toh X.R., Zhang F., Whee D.M., Torta F., Cazenave-Gassiot A., Matsumura T., Kim S. (2017). Mfsd2b Is Essential for the Sphingosine-1-Phosphate Export in Erythrocytes and Platelets. Nature.

[21] Sato K., Malchinkhuu E., Horiuchi Y., Mogi C., Tomura H., Tosaka M., Yoshimoto Y., Kuwabara A., Okajima F. (2007). Critical Role of ABCA1 Transporter in Sphingosine 1-Phosphate Release from Astrocytes. J. Neurochem..

[22] Kase H., Hattori Y., Jojima T., Okayasu T., Tomizawa A., Suzuki K., Banba N., Monden T., Satoh H., Akimoto K. (2007). Globular Adiponectin Induces Adhesion Molecule Expression through the Sphingosine Kinase Pathway in Vascular Endothelial Cells. Life Sci..

[23] Botta A., Elizbaryan K., Tashakorinia P., Lam N.H., Sweeney G. (2020). An Adiponectin-S1P Autocrine Axis Protects Skeletal Muscle Cells from Palmitate-Induced Cell Death. Lipids Health Dis..

[24] Cordeiro A.V., Silva V.R.R., Pauli J.R., da Silva A.S.R., Cintra D.E., Moura L.P., Ropelle E.R. (2019). The Role of Sphingosine-1-Phosphate in Skeletal Muscle: Physiology, Mechanisms, and Clinical Perspectives. J. Cell Physiol..

[25] Donati C., Cencetti F., Bruni P. (2013). Sphingosine 1-Phosphate Axis: A New Leader Actor in Skeletal Muscle Biology. Front. Physiol..

[26] Donati C., Cencetti F., Bruni P. (2013). New Insights into the Role of Sphingosine 1-Phosphate and Lysophosphatidic Acid in the Regulation of Skeletal Muscle Cell Biology. Biochim. Biophys. Acta.

[27] Nagata Y., Partridge T.A., Matsuda R., Zammit P.S. (2006). Entry of Muscle Satellite Cells into the Cell Cycle Requires Sphingolipid Signaling. J. Cell Biol..

[28] Donati C., Meacci E., Nuti F., Becciolini L., Farnararo M., Bruni P. (2005). Sphingosine 1-Phosphate Regulates Myogenic Differentiation: A Major Role for S1P2 Receptor. FASEB J..

[29] Danieli-Betto D., Germinario E., Esposito A., Megighian A., Midrio M., Ravara B., Damiani E., Libera L.D., Sabbadini R.A., Betto R. (2005). Sphingosine 1-Phosphate Protects Mouse Extensor Digitorum Longus Skeletal Muscle during Fatigue. Am. J. Physiol. Cell Physiol..

[30] Rapizzi E., Taddei M.L., Fiaschi T., Donati C., Bruni P., Chiarugi P. (2009). Sphingosine 1-Phosphate Increases Glucose Uptake through Trans-Activation of Insulin Receptor. Cell. Mol. Life Sci..

[31] Formigli L., Francini F., Meacci E., Vassalli M., Nosi D., Quercioli F., Tiribilli B., Bencini C., Piperio C., Bruni P. (2002). Sphingosine 1-Phosphate Induces Ca^2+^ Transients and Cytoskeletal Rearrangement in C2C12 Myoblastic Cells. Am. J. Physiol. Cell Physiol..

[32] Bernacchioni C., Cencetti F., Ouro A., Bruno M., Gomez-Muñoz A., Donati C., Bruni P. (2018). Lysophosphatidic Acid Signaling Axis Mediates Ceramide 1-Phosphate-Induced Proliferation of C2C12 Myoblasts. Int. J. Mol. Sci..

[33] Bruno G., Cencetti F., Bernacchioni C., Donati C., Blankenbach K.V., Thomas D., Meyer Zu Heringdorf D., Bruni P. (2018). Bradykinin Mediates Myogenic Differentiation in Murine Myoblasts through the Involvement of SK1/Spns2/S1P2 Axis. Cell. Signal..

[34] Livak K.J., Schmittgen T.D. (2001). Analysis of Relative Gene Expression Data Using Real-Time Quantitative PCR and the 2(-Delta Delta C(T)) Method. Methods.

[35] Gulbins A., Schumacher F., Becker K.A., Wilker B., Soddemann M., Boldrin F., Müller C.P., Edwards M.J., Goodman M., Caldwell C.C. (2018). Antidepressants Act by Inducing Autophagy Controlled by Sphingomyelin-Ceramide. Mol. Psychiatry.

[36] Naser E., Kadow S., Schumacher F., Mohamed Z.H., Kappe C., Hessler G., Pollmeier B., Kleuser B., Arenz C., Becker K.A. (2020). Characterization of the Small Molecule ARC39, a Direct and Specific Inhibitor of Acid Sphingomyelinase in Vitro. J. Lipid. Res..

[37] Bernacchioni C., Ghini V., Squecco R., Idrizaj E., Garella R., Puliti E., Cencetti F., Bruni P., Donati C. (2021). Role of Sphingosine 1-Phosphate Signalling Axis in Muscle Atrophy Induced by TNFα in C2C12 Myotubes. Int. J. Mol. Sci..

[38] Cencetti F., Bernacchioni C., Bruno M., Squecco R., Idrizaj E., Berbeglia M., Bruni P., Donati C. (2019). Sphingosine 1-Phosphate-Mediated Activation of Ezrin-Radixin-Moesin Proteins Contributes to Cytoskeletal Remodeling and Changes of Membrane Properties in Epithelial Otic Vesicle Progenitors. Biochim. Biophys. Acta Mol. Cell Res..

[39] Squecco R., Carraro U., Kern H., Pond A., Adami N., Biral D., Vindigni V., Boncompagni S., Pietrangelo T., Bosco G. (2009). A Subpopulation of Rat Muscle Fibers Maintains an Assessable Excitation-Contraction Coupling Mechanism after Long-Standing Denervation despite Lost Contractility. J. Neuropathol. Exp. Neurol..

[40] Collins C.A., Rojas E., Suarez-Isla B.A. (1982). Fast Charge Movements in Skeletal Muscle Fibres from Rana Temporaria. J. Physiol..

[41] Squecco R., Chellini F., Idrizaj E., Tani A., Garella R., Pancani S., Pavan P., Bambi F., Zecchi-Orlandini S., Sassoli C. (2020). Platelet-Rich Plasma Modulates Gap Junction Functionality and Connexin 43 and 26 Expression During TGF-Β1-Induced Fibroblast to Myofibroblast Transition: Clues for Counteracting Fibrosis. Cells.

[42] Squecco R., Idrizaj E., Morelli A., Gallina P., Vannelli G.B., Francini F. (2016). An Electrophysiological Study on the Effects of BDNF and FGF2 on Voltage Dependent Ca(2+) Currents in Developing Human Striatal Primordium. Mol. Cell. Neurosci..

[43] Mannelli M., Gamberi T., Magherini F., Fiaschi T. (2021). A Metabolic Change towards Fermentation Drives Cancer Cachexia in Myotubes. Biomedicines.

[44] Takis P.G., Ghini V., Tenori L., Turano P., Luchinat C. (2019). Uniqueness of the NMR Approach to Metabolomics. TrAC Trends Anal. Chem..

[45] Bernacchioni C., Ghini V., Cencetti F., Japtok L., Donati C., Bruni P., Turano P. (2017). NMR Metabolomics Highlights Sphingosine Kinase-1 as a New Molecular Switch in the Orchestration of Aberrant Metabolic Phenotype in Cancer Cells. Mol. Oncol..

[46] D’Alessandro G., Quaglio D., Monaco L., Lauro C., Ghirga F., Ingallina C., de Martino M., Fucile S., Porzia A., Di Castro M.A. (2019). 1H-NMR Metabolomics Reveals the Glabrescione B Exacerbation of Glycolytic Metabolism beside the Cell Growth Inhibitory Effect in Glioma. Cell Commun. Signal..

[47] Ghini V., Senzacqua T., Massai L., Gamberi T., Messori L., Turano P. (2021). NMR Reveals the Metabolic Changes Induced by Auranofin in A2780 Cancer Cells: Evidence for Glutathione Dysregulation. Dalton Trans..

[48] Vignoli A., Ghini V., Meoni G., Licari C., Takis P.G., Tenori L., Turano P., Luchinat C. (2019). High-Throughput Metabolomics by 1D NMR. Angew. Chem. Int. Ed. Engl..

[49] Pitson S.M., Xia P., Leclercq T.M., Moretti P.A.B., Zebol J.R., Lynn H.E., Wattenberg B.W., Vadas M.A. (2004). Phosphorylation-Dependent Translocation of Sphingosine Kinase to the Plasma Membrane Drives Its Oncogenic Signalling. J. Exp. Med..

[50] Hait N.C., Bellamy A., Milstien S., Kordula T., Spiegel S. (2007). Sphingosine Kinase Type 2 Activation by ERK-Mediated Phosphorylation. J. Biol. Chem..

[51] Baskin K.K., Winders B.R., Olson E.N. (2015). Muscle as a “Mediator” of Systemic Metabolism. Cell Metab..

[52] Bernacchioni C., Cencetti F., Blescia S., Donati C., Bruni P. (2012). Sphingosine Kinase/Sphingosine 1-Phosphate Axis: A New Player for Insulin-like Growth Factor-1-Induced Myoblast Differentiation. Skelet. Muscle.

[53] Nincheri P., Bernacchioni C., Cencetti F., Donati C., Bruni P. (2010). Sphingosine Kinase-1/S1P1 Signalling Axis Negatively Regulates Mitogenic Response Elicited by PDGF in Mouse Myoblasts. Cell. Signal..

[54] Donati C., Nincheri P., Cencetti F., Rapizzi E., Farnararo M., Bruni P. (2007). Tumor Necrosis Factor-Alpha Exerts pro-Myogenic Action in C2C12 Myoblasts via Sphingosine Kinase/S1P2 Signaling. FEBS Lett..

[55] Cencetti F., Bruno G., Bernacchioni C., Japtok L., Puliti E., Donati C., Bruni P. (2020). Sphingosine 1-Phosphate Lyase Blockade Elicits Myogenic Differentiation of Murine Myoblasts Acting via Spns2/S1P2 Receptor Axis. Biochim. Biophys. Acta Mol. Cell. Biol. Lipids.

[56] Becciolini L., Meacci E., Donati C., Cencetti F., Rapizzi E., Bruni P. (2006). Sphingosine 1-Phosphate Inhibits Cell Migration in C2C12 Myoblasts. Biochim. Biophys. Acta.

[57] Cencetti F., Bernacchioni C., Tonelli F., Roberts E., Donati C., Bruni P. (2013). TGFβ1 Evokes Myoblast Apoptotic Response via a Novel Signaling Pathway Involving S1P4 Transactivation Upstream of Rho-Kinase-2 Activation. FASEB J..

[58] Cencetti F., Bernacchioni C., Nincheri P., Donati C., Bruni P. (2010). Transforming Growth Factor-Beta1 Induces Transdifferentiation of Myoblasts into Myofibroblasts via up-Regulation of Sphingosine Kinase-1/S1P3 Axis. Mol. Biol. Cell.

[59] Botta A., Liu Y., Wannaiampikul S., Tungtrongchitr R., Dadson K., Park T.-S., Sweeney G. (2019). An Adiponectin-S1P Axis Protects against Lipid Induced Insulin Resistance and Cardiomyocyte Cell Death via Reduction of Oxidative Stress. Nutr. Metab..

[60] Ikeda Y., Ohashi K., Shibata R., Pimentel D.R., Kihara S., Ouchi N., Walsh K. (2008). Cyclooxygenase-2 Induction by Adiponectin Is Regulated by a Sphingosine Kinase-1 Dependent Mechanism in Cardiac Myocytes. FEBS Lett..

[61] Cuvillier O., Pirianov G., Kleuser B., Vanek P.G., Coso O.A., Gutkind S., Spiegel S. (1996). Suppression of Ceramide-Mediated Programmed Cell Death by Sphingosine-1-Phosphate. Nature.

[62] Hait N.C., Oskeritzian C.A., Paugh S.W., Milstien S., Spiegel S. (2006). Sphingosine Kinases, Sphingosine 1-Phosphate, Apoptosis and Diseases. Biochim. Biophys. Acta.

[63] Vasiliauskaité-Brooks I., Sounier R., Rochaix P., Bellot G., Fortier M., Hoh F., de Colibus L., Bechara C., Saied E.M., Arenz C. (2017). Structural Insights into Adiponectin Receptors Suggest Ceramidase Activity. Nature.

[64] Alemany R., van Koppen C.J., Danneberg K., ter Braak M., Meyer zu Heringdorf D. (2007). Regulation and Functional Roles of Sphingosine Kinases. Naunyn-Schmied Arch. Pharmacol..

[65] Laurenzana A., Cencetti F., Serratì S., Bruno G., Japtok L., Bianchini F., Torre E., Fibbi G., Del Rosso M., Bruni P. (2015). Endothelial Sphingosine Kinase/SPNS2 Axis Is Critical for Vessel-like Formation by Human Mesoangioblasts. J. Mol. Med..

[66] Mizugishi K., Yamashita T., Olivera A., Miller G.F., Spiegel S., Proia R.L. (2005). Essential Role for Sphingosine Kinases in Neural and Vascular Development. Mol. Cell. Biol..

[67] Adiponectin Ameliorates Doxorubicin-Induced Cardiotoxicity through Akt Protein-Dependent Mechanism—PubMed. https://pubmed.ncbi.nlm.nih.gov/21784858/.

[68] Sassoli C., Formigli L., Bini F., Tani A., Squecco R., Battistini C., Zecchi-Orlandini S., Francini F., Meacci E. (2011). Effects of S1P on Skeletal Muscle Repair/Regeneration during Eccentric Contraction. J. Cell. Mol. Med..

[69] Tanaka Y., Kita S., Nishizawa H., Fukuda S., Fujishima Y., Obata Y., Nagao H., Masuda S., Nakamura Y., Shimizu Y. (2019). Adiponectin Promotes Muscle Regeneration through Binding to T-Cadherin. Sci. Rep..

[70] Christé G., Bonvallet R., Chouabe C. (2020). Accounting for Cardiac T-Tubule Increase with Age and Myocyte Volume to Improve Measurements of Its Membrane Area and Ionic Current Densities. Prog. Biophys. Mol. Biol..

[71] Yamauchi T., Kamon J., Minokoshi Y., Ito Y., Waki H., Uchida S., Yamashita S., Noda M., Kita S., Ueki K. (2002). Adiponectin Stimulates Glucose Utilization and Fatty-Acid Oxidation by Activating AMP-Activated Protein Kinase. Nat. Med..

[72] Iwabu M., Yamauchi T., Okada-Iwabu M., Sato K., Nakagawa T., Funata M., Yamaguchi M., Namiki S., Nakayama R., Tabata M. (2010). Adiponectin and AdipoR1 Regulate PGC-1alpha and Mitochondria by Ca(2+) and AMPK/SIRT1. Nature.

[73] Inoki K., Kim J., Guan K.-L. (2012). AMPK and MTOR in Cellular Energy Homeostasis and Drug Targets. Annu. Rev. Pharmacol. Toxicol..

[74] Fiaschi T., Tedesco F.S., Giannoni E., Diaz-Manera J., Parri M., Cossu G., Chiarugi P. (2010). Globular Adiponectin as a Complete Mesoangioblast Regulator: Role in Proliferation, Survival, Motility, and Skeletal Muscle Differentiation. Mol. Biol. Cell.

[75] Goto A., Ohno Y., Ikuta A., Suzuki M., Ohira T., Egawa T., Sugiura T., Yoshioka T., Ohira Y., Goto K. (2013). Up-Regulation of Adiponectin Expression in Antigravitational Soleus Muscle in Response to Unloading Followed by Reloading, and Functional Overloading in Mice. PLoS ONE.

[76] Inoue A., Cheng X.W., Huang Z., Hu L., Kikuchi R., Jiang H., Piao L., Sasaki T., Itakura K., Wu H. (2017). Exercise Restores Muscle Stem Cell Mobilization, Regenerative Capacity and Muscle Metabolic Alterations via Adiponectin/AdipoR1 Activation in SAMP10 Mice. J. Cachexia Sarcopenia Muscle.

[77] Singh A.K., Shree S., Chattopadhyay S., Kumar S., Gurjar A., Kushwaha S., Kumar H., Trivedi A.K., Chattopadhyay N., Maurya R. (2017). Small Molecule Adiponectin Receptor Agonist GTDF Protects against Skeletal Muscle Atrophy. Mol. Cell. Endocrinol..

[78] Ito R., Higa M., Goto A., Aoshima M., Ikuta A., Ohashi K., Yokoyama S., Ohno Y., Egawa T., Miyata H. (2018). Activation of Adiponectin Receptors Has Negative Impact on Muscle Mass in C2C12 Myotubes and Fast-Type Mouse Skeletal Muscle. PLoS ONE.

[79] Harada H., Kai H., Shibata R., Niiyama H., Nishiyama Y., Murohara T., Yoshida N., Katoh A., Ikeda H. (2017). New Diagnostic Index for Sarcopenia in Patients with Cardiovascular Diseases. PLoS ONE.

[80] Malik F.A., Meissner A., Semenkov I., Molinski S., Pasyk S., Ahmadi S., Bui H.H., Bear C.E., Lidington D., Bolz S.-S. (2015). Sphingosine-1-Phosphate Is a Novel Regulator of Cystic Fibrosis Transmembrane Conductance Regulator (CFTR) Activity. PLoS ONE.

[81] Rapizzi E., Donati C., Cencetti F., Pinton P., Rizzuto R., Bruni P. (2007). Sphingosine 1-Phosphate Receptors Modulate Intracellular Ca2+ Homeostasis. Biochem. Biophys. Res. Commun..

[82] Yang S.J., Choi J.M., Kim L., Park S.E., Rhee E.J., Lee W.Y., Oh K.W., Park S.W., Park C.-Y. (2014). Nicotinamide Improves Glucose Metabolism and Affects the Hepatic NAD-Sirtuin Pathway in a Rodent Model of Obesity and Type 2 Diabetes. J. Nutr. Biochem..

[83] Goody M.F., Henry C.A. (2018). A Need for NAD+ in Muscle Development, Homeostasis, and Aging. Skelet. Muscle.

