# Supplementary material for: S1P Signalling Axis Is Necessary for Adiponectin-Directed Regulation of Electrophysiological Properties and Oxidative Metabolism in C2C12 Myotubes
Source: Cells. 2022 Feb 17;11(4):713. doi: 10.3390/cells11040713 (PMC8869893; doi:10.3390/cells11040713)
Supplement: Supplementary file 1 [file cells-11-00713-s001.zip › cells-1541998-supplementary.pdf]

Supplementary Materials

S1P signalling axis is necessary for Adiponectin-directed regulation of electrophysiological properties and oxidative metabolism in C2C12 myotube

Caterina Bernacchioni[1], Roberta Squecco[2], Tania Gamberi[1], Veronica Ghini[3], Fabian Schumacher[4], Michele Mannelli[1], Rachele Garella[2], Eglantina Idrizaj[2], Francesca Cencetti[1], Elisa Puliti[1], Paola Bruni[1], Paola Turano[3], Tania Fiaschi[1] and Chiara Donati[1,*]

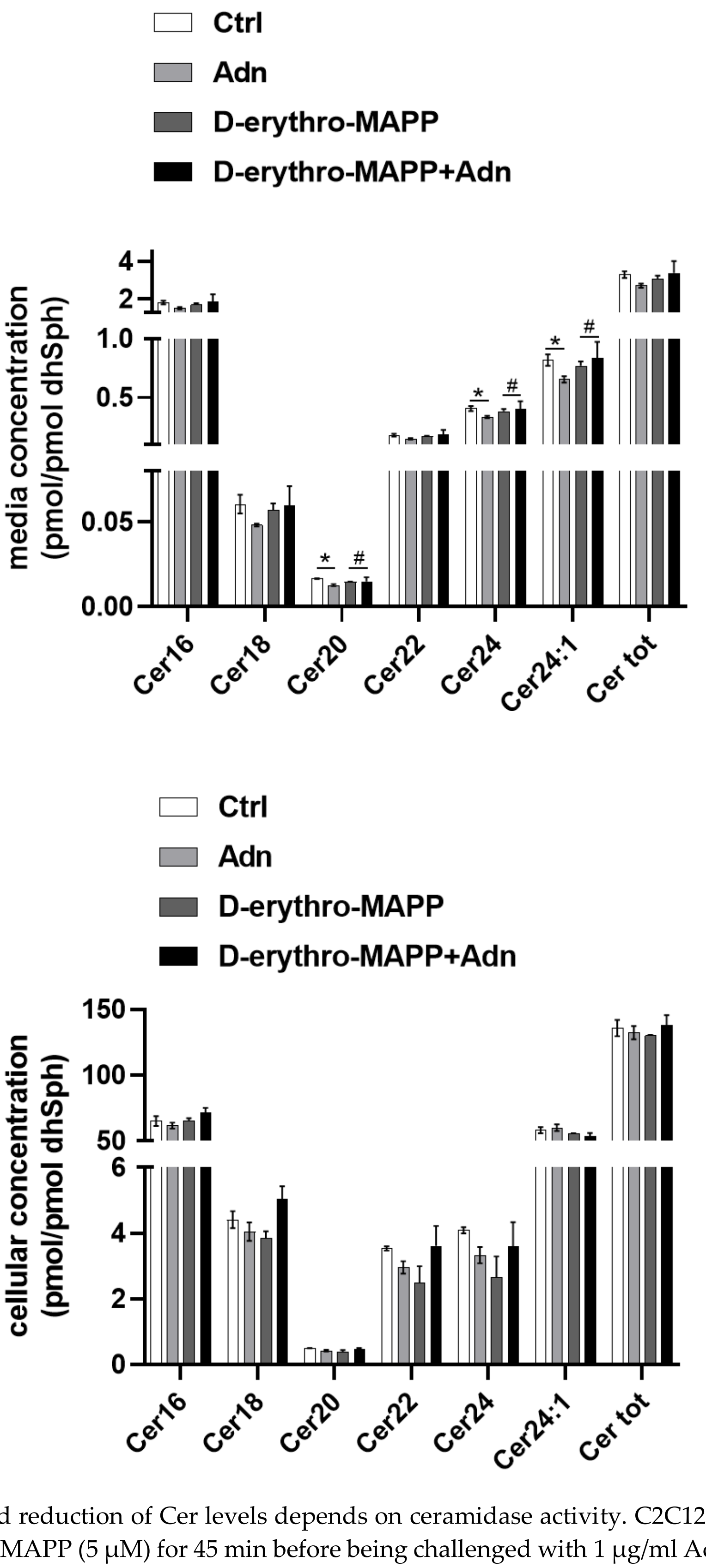

Figure S1. Adn-induced reduction of Cer levels depends on ceramidase activity. C2C12 myotubes were pre-treated with D-erythro-MAPP (5 μM) for 45 min before being challenged with 1 μg/ml Adn for 1 h. The media were collected (upper panel) and cells harvested (lower panel) and then subjected to Cer analysis. The decrease of Cer20, Cer24 and Cer24:1 levels induced by Adn was statistically significant by Student's t-test *$p<0.05$. The effect of D-erythro-MAPP is statistically significant by two-way ANOVA followed by Bonferroni’s post hoc test (#$p > 0.05$).

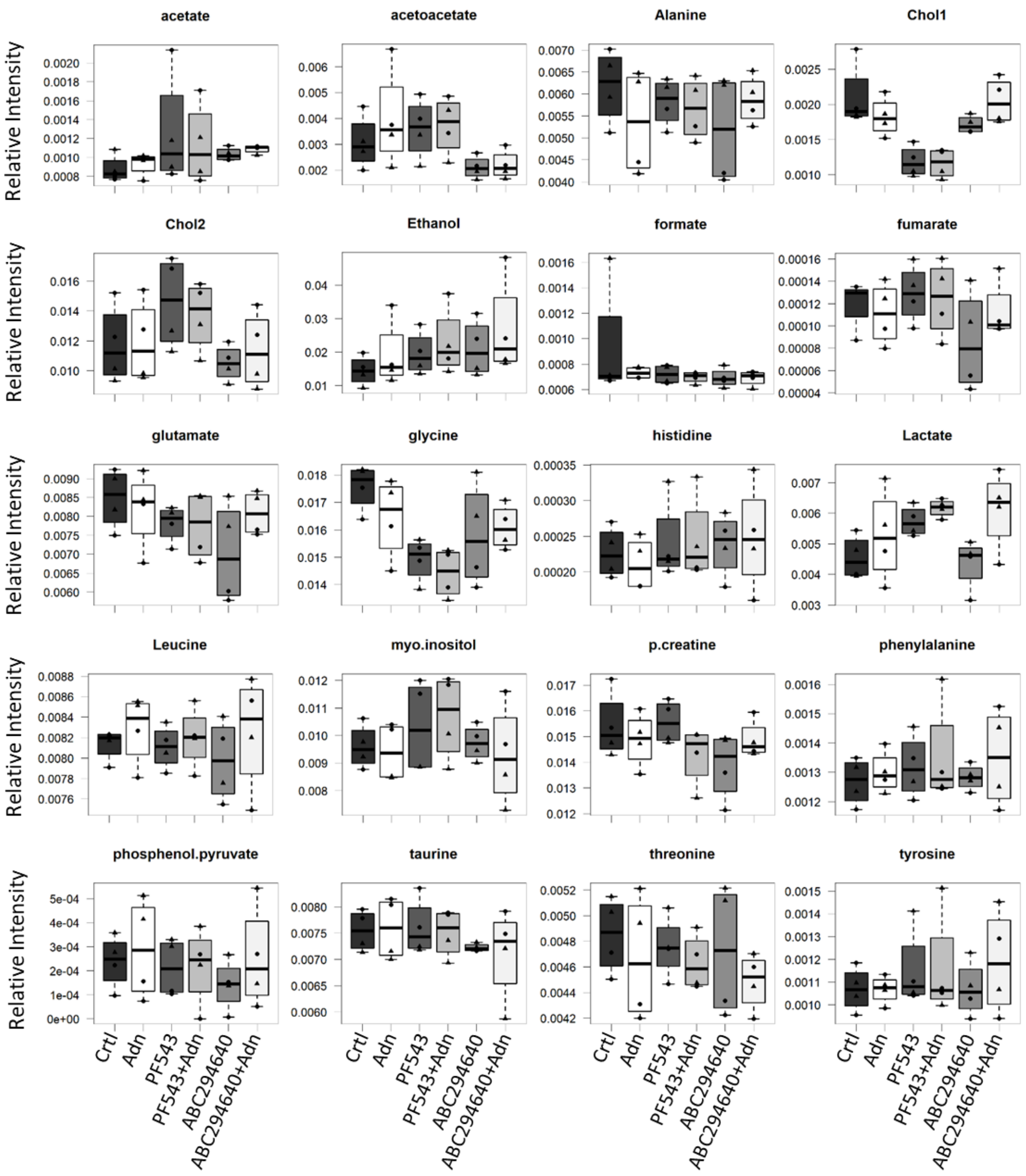

Figure S2. ^{1}H-NMR-based endometabolome profiles. C2C12 myotubes were pre-treated with specific SK1 inhibitor PF543 (10 μM) or SK2 inhibitor ABC294640 (1 μM) for 1 h before being challenged with 1 μg/ml Adn for 24 h. Boxplot representing metabolites levels, in cell lysates, in the different group of samples.

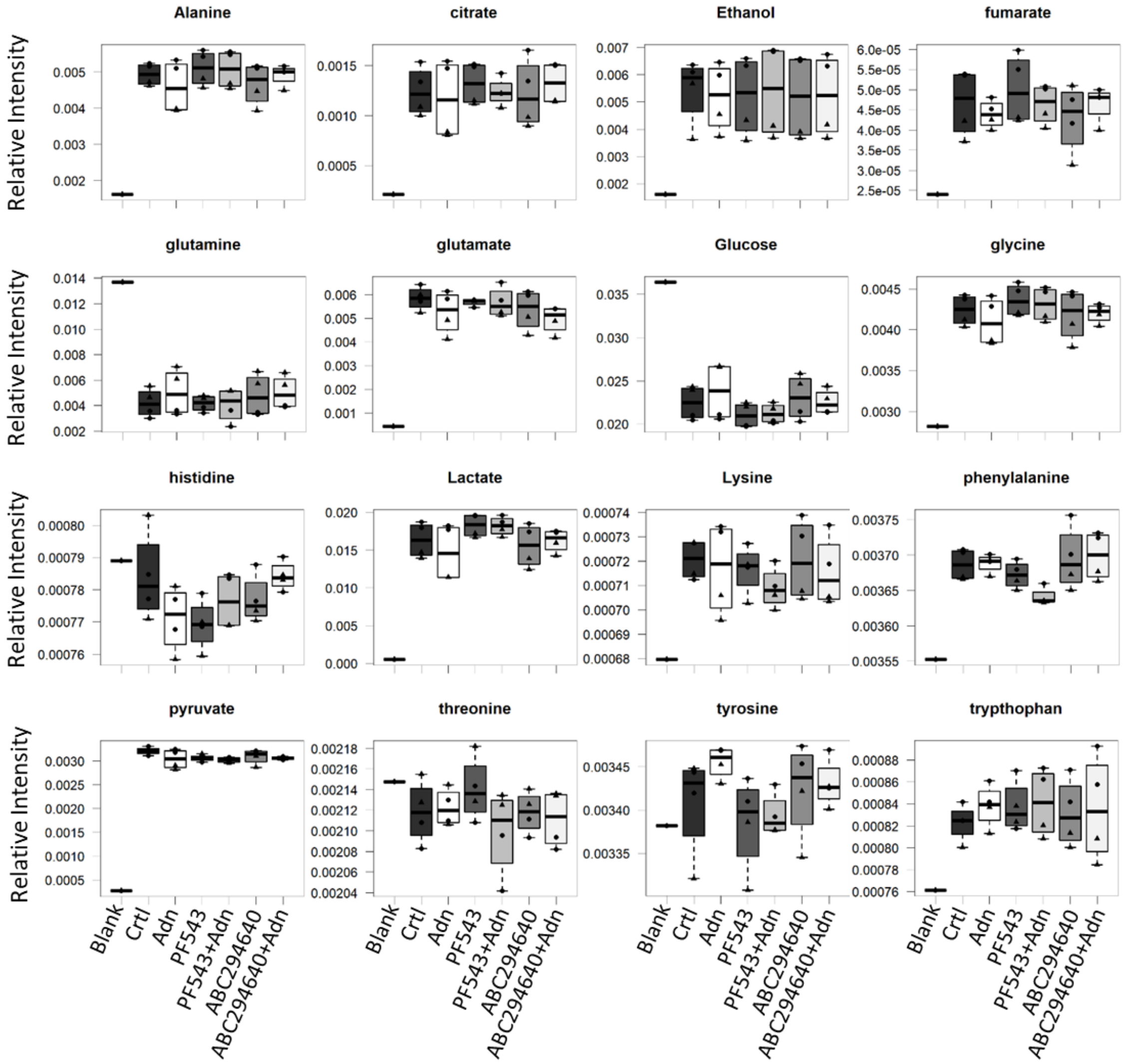

Figure S3. ^{1}H-NMR-based exometabolome profiles. C2C12 myotubes were pre-treated with specific SK1 inhibitor PF543 (10 µM) or SK2 inhibitor ABC294640 (1 µM) for 1 h before being challenged with 1 µg/ml Adn for 24 h. Boxplot representing metabolites levels, in conditioned media, in the different group of samples.
